# Genetic medicines for epilepsy: unlocking new avenues for seizure control

**DOI:** 10.3389/fbioe.2026.1768550

**Published:** 2026-04-24

**Authors:** Ing Chee Wee, Magdalena Przybyla, Jamelle Touma, Lars Matthias Ittner, Janet van Eersel

**Affiliations:** Dementia Research Centre, Macquarie Medical School, Faculty of Medicine, Health and Human Sciences, Macquarie University, Sydney, Australia

**Keywords:** epilepsy, gene editing, gene therapy, genetic medicines, seizures

## Abstract

Epilepsy affects millions of people globally and is marked by unpredictable seizures due to excessive brain activity. These seizures not only vary widely in brain origin and severity but can also be associated with a range of factors - from head injuries to infections to genetic causes. Although antiseizure medications provide effective seizure control for many patients, approximately 30% experience drug-resistant epilepsy, with syndromic forms such as Lennox-Gastaut and Dravet syndrome posing significant therapeutic challenges. In addition, current pharmacological treatments are often associated with significant side effects and typically do not address the underlying pathophysiology. Gene therapies and genetic medicines are groundbreaking treatment modalities that enable direct targeting of disease mechanisms and associated genes. After FDA approval of the very first gene therapy in 2017 and the discovery of CRISPR-based gene editing, the field has rapidly expanded offering new hope for epilepsy treatment. This review highlights the latest advancements and therapeutic approaches for genetic medicines and explores their potential to transform the therapeutic landscape of epilepsy.

## Graphical Abstract

**Figure:**
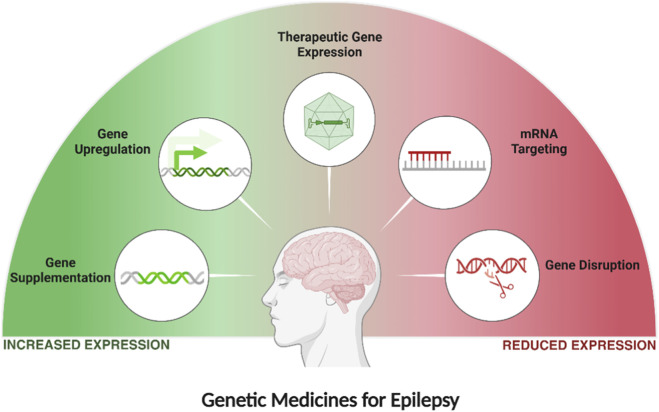

## An introduction to epilepsy

Epilepsy refers to a group of heterogeneous neurological diseases that are characterized by an enduring predisposition to seizures, caused by excessive neuronal brain activity (Fisher et al., 2014). It affects over 50 million individuals across all ages, ethnicities, and geographical locations, making it one of the most common neurological conditions (Fiest et al., 2017; Ngugi et al., 2010). The main characteristic of epilepsy is seizures, although their origin, frequency, and severity can vary widely between patients, reflecting its complex aetiology (Thomas and Berkovic, 2014). Seizures can be classified according to their onset, which can be focal (impacting a specific brain region), generalized (involving both hemispheres), or of unknown nature (the origin cannot be identified) (Devinsky et al., 2018). Among these, focal seizures are the most common, with a substantial proportion originating in the temporal lobe, referred to as temporal lobe epilepsy (Kotsopoulos et al., 2002).

In most patients, the underlying cause of epilepsy remains elusive, which is further complicated by the fact that the condition can arise from virtually any insult that disrupts normal brain function (Devinsky et al., 2018). Genetic factors also play a critical role, ranging from monogenic mutations and polygenic risk to complex gene-gene and gene-environment interactions. To date, over 900 genes have been linked to epilepsy (Devinsky et al., 2018), many of which are involved in regulating processes such as neuronal excitability, cell signalling, synapse formation, and neuronal metabolism. Remarkably, mutations in the same gene can lead to vastly divergent clinical presentations. Of particular interest, pathogenic variants in genes encoding ion-channel subunits have been implicated in a spectrum of developmental and epileptic encephalopathies (DEEs). Among these, *de novo* mutations in the *SCN1A* gene, which encodes the voltage-gated NaV1.1 sodium channel, are causally involved in the development of Dravet syndrome (DS), a severe epileptic encephalopathy with onset in early childhood (Claes et al., 2001; Nakahara Sakamoto et al., 2025).

When provided with an accurate diagnosis and appropriate treatment, approximately 70% of patients can achieve seizure-free status (Manole et al., 2023; Hersi et al., 2022). The most common treatment option, pharmacological interventions known as antiseizure medications (ASMs), are generally effective in reducing seizure frequency and severity (Milligan, 2021). However, around 30% of patients experience drug-resistant epilepsy, often referred to as refractory or intractable epilepsy (Tang et al., 2017). Certain DEEs, such as Lennox-Gastaut syndrome and DS, are prototypical examples in which complex underlying pathophysiology contributes to their resistance to conventional ASMs. These patients often require polytherapy, leading to an increased risk of adverse effects and complicated long-term management. ASMs are commonly associated with numerous adverse effects, including headaches, fatigue, dizziness, blurred vision, weight changes, and mood alterations, which can place an additional economic burden on patients (Mutanana et al., 2020). Unfortunately, the current treatments typically do not address the root cause of seizures. It is therefore essential to continue developing more effective treatments that can minimize the physiological and socio-economic impact of epilepsy.

## The emergence of gene therapy and genetic medicines

Gene therapies have emerged as a promising new avenue for a wide range of conditions previously considered untreatable via traditional approaches (High and Roncarolo, 2019). These therapies aim to deliver genetic material into target cells to ultimately modify their biological properties for a therapeutic purpose. Through this approach, gene therapies can be used to directly (or indirectly) target causative disease mechanisms and can be engineered to specifically manipulate desired target cells, potentially minimizing off-target effects (Weinberg and McCown, 2013). Currently, over three dozen approved gene therapies are available, including treatments for sickle cell disease, inherited retinal disorders, and various cancers, making them one of the biggest success stories of the 21st century (Administration, 2025).

**FIGURE 1:**
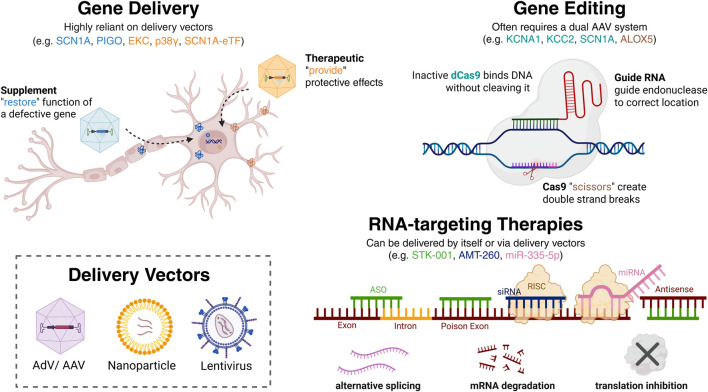
Summary of current therapeutic strategies by which gene therapies and genetic medicines can be utilized for the treatment of epilepsy. Gene delivery involves the provision of exogenous genetic material with the aim of either compensating for the malfunction of the native gene (Supplement) or expressing a therapeutic gene to promote cell survival (Therapeutic). Gene editing involves the direct targeting of genomic DNA sequences (e.g., with dCas9 or Cas9) with the aim of repairing, enhancing or disrupting gene expression. Gene expression can also be modulated with RNA-targeting therapies, by targeting alternative splicing, enhancing mRNA degradation, or inhibiting RNA translation. Generally, these methods aim to either increase or decrease the expression of a target gene(s), depending on the therapeutic goal. Various delivery vectors can be utilized to apply these genetic medicines, including viral vectors and nanoparticles. Together, these approaches represent a growing toolkit for the precision treatment of diverse forms of epilepsy. AAV, adeno-associated virus; AdV, adenovirus; ASO, antisense oligonucleotide; miRNA, microRNA; RISC, RNA-Induced Silencing Complex; siRNA, short interfering RNA. Created in http://Biorender.com.

Historically, gene therapy was simply defined as the delivery and transfer of genetic material into a specific target cell (Mulliga and n, 1993). However, with recent advances, this definition has expanded to include genome-editing medicines and nucleic acids that can be utilized to regulate, replace, modulate, or even introduce new genetic sequences (von Fritschen et al., 2024). Furthermore, therapeutic application may involve an *ex vivo* approach, where the patient’s cells are genetically modified outside the body before being reintroduced, or an *in vivo* approach, where the therapy is administered directly to the patient (e.g., intravenously), enabling gene modifications directly within the body. This review will explore recent advances in the field of gene therapies and genetic medicines (see Figure 1), highlighting a range of gene delivery vehicles, emerging therapeutic strategies, and key findings from both pre-clinical and clinical studies investigating innovative treatment options for epilepsy.

## Delivery methods for genetic medicines

A critical challenge for genetic therapies lies in the effective delivery of genetic material to the target cells. In neurological conditions, this is further complicated by the need to cross the blood-brain barrier (BBB) (Bellettato et al., 2018). Currently, engineered viruses in which native disease-causing genes are replaced with therapeutic sequences are the predominant vehicle of choice for gene therapy delivery, leveraging their natural ability to efficiently deliver their genome into host cells (Sandoval-Villegas and Ivics, 2024; Lundstrom, 2018). In addition, extensive research has gone into developing non-viral vectors to help overcome some of the issues associated with viral-based delivery methods (Guan X. et al., 2024).

**Adeno-associated virus (AAV)** vectors are known for their safety profile, potential for persistence in episomal form, and ability to provide long-term therapeutic gene expression (Batty and Lillicrap, 2024). However, a significant limitation associated with AAV vectors is their relatively small packaging capacity (∼4.7kb, including two critical inverted terminal repeat [ITR] sequences flanking each end of the desired gene cassette), making them unsuitable for lengthier genetic sequences (Benatti and Gray-Edwards, 2022). This restriction poses a particular hurdle for epilepsy, as many candidate interventions require genetic sequences that exceed the size limit of AAV vectors. Additionally, targeting specific brain regions using AAV can be challenging, and high doses may be required for effective delivery, potentially increasing the risk of an immune response (Ronzitti et al., 2020). It should be noted that AAVs can be classified into different serotypes (1–13, as well as many subvariants), each with unique properties (Issa et al., 2023). For instance, AAV9 efficiently crosses the BBB and is therefore widely used for neurological disorders, while AAV8 exhibits strong liver tropism and is therefore often employed for metabolic and hematologic conditions (Zhao et al., 2024). AAV serotype selection is therefore critically important for successful clinical application. Scientists have also engineered additional variants (e.g., PhP.eB and CAP-B10) derived from AAV9 that were optimised for greater transduction efficiency and specificity for astrocytes and neurons in the central nervous system (Deverman et al., 2016; Goertsen et al., 2022).

The efficacy of AAV serotypes depends not only on the virus’s ability to enter the target cell but also on the speed with which the delivered genetic material can be converted into a transcriptionally active sequence (Pupo et al., 2022). With traditional AAV, single-stranded DNA packaged within the virus is transcriptionally inert and must first be converted to double-stranded DNA before transcription can occur (Wang et al., 2019). In contrast, self-complementary AAV (scAAV) utilizes a modified ITR sequence that enables complementary single-stranded DNA sequences to quickly self-anneal after the virus is uncoated and is thus readily double-stranded by design (Zhang et al., 2024). These scAAV sequences can therefore undergo transcription immediately and have been shown to enhance transduction efficiency compared to conventional AAV sequences (McCarty et al., 2001). Already, a next-generation scAAV that relies on covalently closed-ends (cceAAV) rather than modified ITRs has been developed and successfully trialled in human patients (Duan et al., 2025). With further refinement, it is anticipated that AAV-based therapies will give rise to a new standard of treatment care for epilepsy and other diseases (Pupo et al., 2022).

**Lentiviruses (LVs)** are single-stranded RNA viruses that are equipped with a reverse transcriptase enzyme that can convert RNA into DNA (Bulcha et al., 2021) and stably integrate genetic sequences into the host’s genome. This enables them to effectively deliver genetic material to both dividing and non-dividing cells, offering a versatile option for gene therapy delivery (Osten et al., 2006). LVs are particularly beneficial in terms of packaging capacity, with a significantly larger limit of ∼9 kb (Assaf et al., 2023). However, insertional mutagenesis is a major safety concern with LVs, where random integration of the delivered transgene disrupts a critical gene(s) (Escors and Breckpot, 2010). Further, they are known to induce a greater immune response than AAVs, which can be potentially harmful (Shirley et al., 2020). However, optimization of the LV vector system has mitigated some risks, leading to the approval of *ex vivo* LV-based gene therapies for several disorders (Kingwell, 2022), with several others currently undergoing clinical assessment (Davies et al., 2024).

**Adenoviruses (AdVs)** were one of the most utilized vectors in clinical trials until the rise in popularity of AAVs (Bulcha et al., 2021). They offer distinct advantages, such as a large packaging capacity (∼36 kb), relatively easy large-scale manufacture, good transduction efficacy, and episomal maintenance of the transgene (Leikas et al., 2023). However, a major concern is the emergence of replication-competent AdVs during production, which can have harmful effects (Bulcha et al., 2021; Lion, 2014). Although the AdV primarily targets the respiratory tract, they have a broad tropism profile and evidence suggests that they can be used to target the brain (Barkats et al., 1998; Navarro et al., 1999). Unfortunately, AdV vectors are associated with strong immune responses in humans, significantly limiting their therapeutic use (Sallard et al., 2023).

**Nanoparticles** are emerging as a promising, non-viral delivery system for genetic therapies due to their adaptable size, shape, and biological profile (Mendes et al., 2022). In fact, various types of nanoparticles have already been developed (although mostly at the preclinical stage), including systems based on lipids, polymers, peptides, and inorganic compounds. Notably, most studies utilizing nanoparticles for epilepsy have primarily focused on delivering ASMs rather than exploring their use for genetic medicines (Meng et al., 2022; Zybina et al., 2018; Hou et al., 2022). It is also important to note that issues such as biodegradation, nonspecific adsorption, and ineffective internalization still need to be addressed before they can be broadly applied (Chen et al., 2016).

**Antisense Oligonucleotides (ASOs)** are single-stranded synthetic RNA or DNA sequences (typically ∼12–30 nucleotides) designed to bind to target RNA via complementary base-pairing and thereby modulate gene expression without the need for a vector (Bennett et al., 2019). This can be achieved via degradation of the target RNA, steric hindrance, altering RNA splicing, or by targeting sites critical for translation (Quemener et al., 2020). ASOs offer several advantages compared to viral-based approaches - their inherent degradability and short half-lives (ranging from weeks to months, depending on formulation) allow for adjustable dosing and reversible effects, reducing the risk of prolonged off-target effects. At the same time, chemical modifications, such as 2′O-Methoxyethyl, locked nucleic acid, or phosphorothioate, can be introduced to enhance stability, binding affinity, and nuclease resistance when longer-lasting effects are desired, thereby broadening their therapeutic versatility. For improved brain targeting, ASOs can be delivered directly to the central nervous system via intrathecal injection (Passini et al., 2011). This approach has been successfully employed for the ASO therapeutic Nusinersen to treat spinal muscular atrophy. However, intrathecal delivery can result in uneven drug administration (much of the injected dose remains localized at the injection site) and potential safety concerns due to high local concentration (Mortberg et al., 2023; Rigo et al., 2014; Sumner and Miller, 2024). For this reason, several strategies have been employed to enhance BBB penetrance of ASOs after systemic delivery. Notably, Denali Therapeutics has developed an oligonucleotide transport vehicle (OTV) that binds to transferrin receptor 1, facilitating intravenous delivery and uniform distribution throughout the brain (Barker et al., 2024). Other approaches include the use of nanocarriers, cell-penetrating peptides, and chemical modifications to enhance ASO stability and cellular uptake. These innovations are expanding the therapeutic potential of ASOs for neurological diseases and may reduce the need for invasive administration routes in the future. Encouragingly, over 15 ASOs have already received regulatory approval for a range of conditions, highlighting their therapeutic potential (Roberts et al., 2020).

In summary, although various delivery vehicles and methods are available, these still require further refinement to ensure that genetic medicines can reach their full therapeutic potential. The delivery method of choice will be highly influenced by the therapy’s intended target and mechanism of action. This will then influence treatment efficacy, dosing (i.e., once-off injection *versus* multiple administrations), potential side effects, the permanence of modifications, treatment costs, *etc.* As such, continued development and diversification of effective delivery strategies is essential—not only to advance scientific progress but also to improve patient outcomes.

## How to best utilize gene therapies and medicines for epilepsy?

**FIGURE 2:**
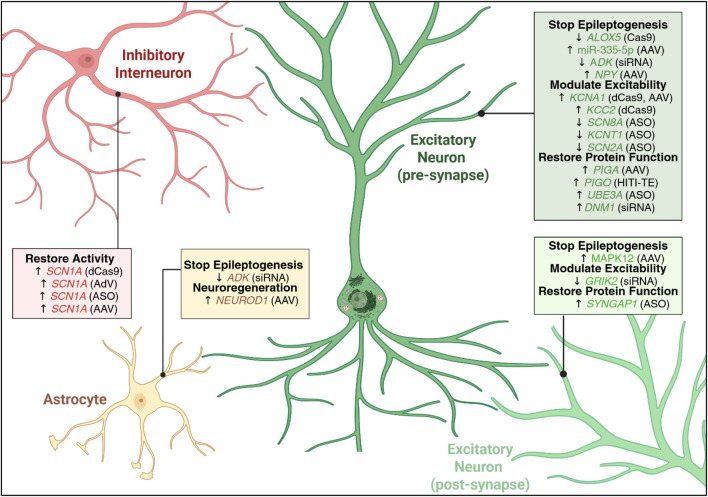
Summary of key genes targeted by current genetic medicines for the treatment of epilepsy. A diverse set of genetic targets has been identified and validated for the development of genetic medicines to treat epileptic disorders. These therapies exert their effects by either upregulating (indicated by upward arrow) or downregulating (indicated by downward arrow) the target gene using a range of therapeutic tools (indicated in brackets). These gene targets are associated with a range of cellular processes - including epileptogenesis, regulation of neuronal excitability, and neuroregeneration - and span multiple cell types, including distinct neuronal subtypes and astrocytes. AAV, adeno-associated virus; AdV, adeno virus; ASO, anti-sense oligonucleotide; dCas9, dead CRISPR Cas9; HITI-TE, homology-independent targeted integration assisted by low-level transgene expression; siRNA, small interfering RNA. Created in https://BioRender.com.

The complex aetiology of epilepsy - ranging from genetic predisposition to acquired disease - presents a wide range of potential targets for therapeutic development (see Figure 2). Although an approved genetic medicine for epilepsy is not yet available, several ongoing clinical trials are underway (summarized in Tables 2 and 3). While these therapies utilize various strategies, all approaches generally aim to restore the balance between excitatory and inhibitory neuronal circuits to achieve therapeutic efficacy. Depending on their target, some of these gene therapies may be applicable to a broad range of epilepsy subtypes, whereas others have been developed for a specific epileptic condition. In the following, we will discuss findings that highlight potential targets and various treatment strategies that are of particular interest to the epilepsy field (summarized in Tables 1–3).

**TABLE 1:** Summary of key studies using gene supplementation and gene editing to treat epileptic disorders.

Strategy: gene supplementation							
Therapy target	Vector	Prom.	Gene	Del.	Preclinical model	Outcomes	References
SCN1A	Adv	*CAG*	*SCN1A*	IC	*Scn1a^+/A1783V^* mice	Administered treatment protected from sudden death, attenuated epileptic seizures, improved motor skills, and greater environmental interaction. However, hyperactivity continued, and cognitive performance varied across tests.	Mora-Jimenez et al. (2021)
		*DP3V*	*SCN1A*	Hip and thalamus	*Scn1a^+/A1783V^* mice	Improved survival and amelioration of the epileptic phenotype in a mouse model of DS.	Ricobaraza et al. (2023)
	AAV2/ PHP.eB	*DLX2.0*	Split-intein *SCN1A*	ICV	*Scn1a* ^*fl*/+^; *Meox2-Cre* DS mice	Prevented post-natal mortality and reduced seizure activity.	Mich et al. (2025)
	AAV PHP.eB	*hSyn*	Cre-loxP *SCN1A* recombination	RO	*Scn1a* ^+/−^	Enhanced survival, restored hippocampal neuronal activity, and reduced seizure-related electrocorticography activity patterns.	Lin et al. (2026)
GPI-anchor biology	AAV PHP.eB	*CAG*	h*PIGA*	IV	*Piga*-floxed mice (Male, *Piga* ^−^) (Female, *Piga* ^+/−^)	Increased expression of hPIGA in the brains significantly extended animal survival (females longer than males); however, strong CAG promoter led to liver impairments.	Murakami et al. (2024)
	AAV PHP.eB	*nEF*	Cas9	IV	*Pigo* KI *Pigo* ^*T130N/T130N*^ mice *Pigo* KIKO *Pigo* ^*T130N/-*^ mice	Increased growth, improved motor ability, reduced seizure susceptibility.	Kuwayama et al. (2022)
	AAV PHP.eB	*U6*	HITIgRNA-*Pigo* cDNA				

Strategy: gene editing							
Therapy target	Vector	Prom.	Gene/CRISPR Enzyme	Del.	Preclinical Model	Outcomes	References
5-lipoxygenase (Alox5)	AAV9	*hSyn*	Cas9	Hip	Pilocarpine (i.p) mice	Treatment reduced seizure severity, delayed epileptic progression, improved epilepsy-associated neuropsychiatric comorbidities (anxiety, cognitive deficit and autistic-like behaviours) and reversed neuronal loss, neurodegeneration, astrogliosis and mossy fiber sprouting.	Guan et al. (2024b)
		*U6*	gRNA-*Alox5*		KA (i.p) and KA (hip) mice		
Potassium channel Kv1.1	AAV9	*Tet-On*	dCas9-VP64	Hip	KA (amygdala) mice	Reduced seizure frequency and increased cognitive performance.	Colasante et al. (2020a)
	AAV9	*U6*	gRNA-*Kcna1*				
K-Cl cotransporter isoform (KCC2)	AAV	*CAMK2A*	Split-intein-dCas9	Subicular	Rapid hippocampal kindling mice model	Upregulation of KCC2 levels alleviated the severity of hippocampal seizures, improved EEG measures and enhanced the anti-seizure effects of diazepam.	Shi et al. (2023)
	AAV	*U6*	gRNA-*KCC2*		KA (hip) mice	KCC2 upregulation reduced diazepam-resistant seizure duration, alleviated valproate-resistant spontaneous seizures and partially restored GABA_A_-mediated inhibition.	
NaV1.1	AAV9	*TRE*	dCas9-VP64	ICV	*Scn1a* ^+/−^ mice	Increased Nav1.1 expression during development is associated with reduced seizure susceptibility.	Colasante et al. (2020b)
	AAV9	*U6*	sgRNA-*Scn1a* promoter region				

AAV, adeno-associated virus; AAV PHP. eB, enhanced neurotrophic variant of AAV9; AdV, adenovirus; CAG, hybrid promoter of cytomegalovirus early enhancer/first intron of chicken β-actin promoter/rabbit β-globin splice acceptor; CAMK2A, Calcium/Calmodulin-Dependent Protein Kinase II Alpha promoter; Del., delivery route; DLX2.0, concatenated core sequence elements of the hDLXI5/6i enhancer; DPV3, a promoter developed based on the Distal-less homolog enhancer, the vesicular GABA transporter promoter and a portion of SCN1A gene; DS, Dravet syndrome; gRNA guide ribonuclear acid; HIP, hippocampal; HITI, Homology-independent targeted integration; hSyn, human synapsin promoter; IC, intracerebral injection; ICV, intracerebroventricular injection; RO, retro orbital injection; IV, intravenous injection; KA, kainic acid; mDlx5/6, GABAergic neuron-specific enhancer; nEF, hybrid EF1α/HTLV promoter; Prom., promoter; SE, status epilepticus; Tet-On, doxycycline induced expression system; TLE, temporal lobe epilepsy; TRE, tetracycline response element; U6, RNA polymerase III U6 promoter; VP64, tetrad repeat of the herpes simplex virus V16 transactivation domain.

**TABLE 2:** Summary of key studies using RNA-targeting therapies to treat epileptic disorders.

Strategy: mRNA targeting (with ASOs)						
Therapy target	Vector	ASO target	Del.	Preclinical Model	Outcomes	References
SCN1A	ASO	STK-001/Zorevunersen prevent inclusion of the nonsense-mediated decay (poison) exon 20N in SCN1A	*ICV*	*Scn1a* ^+/−^ mice	Treatment increased full-length *SCN1A* mRNA and concomitant NaV1.1 protein expression, improving the survival and seizure severity in DS mice. Currently, in phase 3 clinical trial-EMPEROR, which aims to evaluate the effectiveness and safety of STK-001/Zorevunersen in patients with DS.	Han et al. (2020), Wengert et al. (2022), Meglio (2023)
		Surrogate of STK-001 ASO-84			ASO-84 restored action potential firing, increased *SCN1A* mRNA and NaV1.1 expression, as well as GABAergic signalling in parvalbumin-positive interneurons affected by DS, and improved overall survival.	Yuan et al. (2024)
SCN2A	Gapmer-ASO	PRAX-222/Elsunersen Target SCN2A to downregulate NaV1.2 expression	IT	-	Ongoing phase 1/2 clinical trial-EMBRAVE investigating the effect of ASO PRAX-222/Elsunersen on *SCN2A* expression in paediatric participants (aged 2–18) with early-onset SCN2A DEE.	Li et al. (2021)
SCN8A	modified Gapmer-ASO	Target sequence proximal to the 3′ UTR	*ICV*	DEE mouse model Cre-dependent expression of *SCN8A* mutation R1872W	Early treatment reduced *Scn8a* transcript, delayed seizure onset, and lethality in both *SCN8A* encephalopathy and Dravet syndrome mouse models.	Lenk et al. (2020)
				*Scn1a* ^+/−^ mice		
				DEE mouse model Cre-dependent loss of function *Kcnq2*	Repeated treatment of *Scn8a*-ASO reduced *Scn8a* expression, extended the survival of *Kcna1* and *Kcnq2* mutants, and decreased seizure frequency in *Kncq2* mutant mice.	Hill et al. (2022)
				DEE-like mouse model *Kcna1* ^−/−^		
KCNT1	ASO	Target mRNA at the 3′ UTR position	*ICV*	EIMFS model *Kcnt1* P924L mutant	Both early and late treatment showed a significant reduction in seizure frequency, improved behavioural abnormalities, and extended overall survival compared to control-treated animals ASO.	Burbano et al. (2022)
				DEE model Cre-dependent expression of *SCN8A* mutation R1872W	KCNT1-ASO treatment increased survival in both *Scn1a* and *Scn8a* mutant mice.	Hill et al. (2023)
				*Scn1a* ^+/−^ mice		
SynGAP1	SSO	Target alternative 3′ splice site (A3SS) of SynGAP1 intron 10 to prevent nonsense-mediated mRNA decay	-	SynGAP1 patient-derived iPSCs and human iPSC-derived cerebral organoids	Increased SynGAP1 expression.	Yang et al. (2023a)
UBE3A	modified Gapmer ASO	GTX-102 Targets cluster 2 of SNORD115 to remove repression of *UBE3A*	IT	Angelman syndrome iPSC and cynomolgus macaques	Aims to restore expression of UBE3A. Currently moved into phase 3 clinical trial-ASPIRE to evaluate the effect of GTX-102 on cognitive function in Angelman syndrome participants.	Dindot et al. (2023), Milazzo et al. (2021), Meng et al. (2015)

Strategy: mRNA TARGETING (with AAVs-Micro/siRNA)							
Therapy target	Vector	Promoter	Gene	Del.	Preclinical Model	Outcomes	References
SCN1A	AAV9	*hSyn*	miR-335-5p	Hip	PTZ (i.p) mice	AAV injections reduced seizure susceptibility and improved survival rates.	Heiland et al. (2023)
ADK	AAV9	*GFAP*	miR-ADK	Hip	KA (Hip) rat	AAV treatment led to reduced seizure durations and attenuated neuronal cell loss in the hippocampus.	Young et al. (2014)
DNM1	scAAV9	*U6*	miR-Dnm1a	ICV	DEE-like mouse model *Dnm1* ^ftfl/ftfl^	Silencing of a specific *Dnm1a* isoform reduced seizure severity, extended lifespan, promoted growth, rescued several motor impairments, and normalized gliosis and cellular degeneration.	Aimiuwu et al. (2020)
GluK2	AAV9	*hSyn*	AMT260 miR-GRIK2	Hip	Pilocarpine (i.p) mice. TLE patient’s Hip organotypic slice	Silencing GluK2 expression inhibited chronic seizures in mouse models, potentially halting cognitive decline and reducing hyperactivity. This gene therapy progressed to a GenTLE Phase 1/2 clinical trial to investigate its effects in adults with MTLE.	Boileau et al. (2023), Baudouin et al. (2024)

3′UTR, untranslated region; AAV, adeno-associated virus; ASO, antisense oligonucleotide; DEE, developmental and epileptic encephalopathies; Del., delivery route; DS, dravet syndrome; EIMFS, early infantile myoclonic epilepsy syndrome; GFAP, glial fibrillary acidic protein (astrocyte-specific) promoter; HIP, hippocampal; hSyn, human synapsin promoter; ICV, intracerebroventricular injection; i. p., intraperitoneal injection; iPSC, induced pluripotent stem cells; IT, intrathecal injection; KA, kainic acid; miR, microRNA; MTLE, mesial temporal lobe epilepsy; PTZ, pentylenetetrazol; scAAV, self-complementary AAV; SSO, splice switching oligonucleotide; U6, RNA polymerase III promoter.

**TABLE 3:** Summary of key studies using therapeutic gene therapies to treat epileptic disorders.

Strategy: therapeutic gene expression							
Therapy target	Vector	Promoter	Gene	Del.	Preclinical model	Outcomes	References
Potassium channel Kv1.1	LV	*CMV*	*KCNA1*	RMC	Tetanus toxin (intra-motor cortex) rats	Co-injection of tetanus toxin and Kv1.1 LV suppressed seizure activity and prevented epilepsy development.	Wykes et al. (2012)
	LV	*CMV/CAMK2A*	EKC *KCNA1*/EKC	Visual cortex	Tetanus toxin (visual cortex) rats	V1 injections reduced seizure frequency and suppressed focal neuronal excitability. Currently moved to clinical trial phase 1/2 to assess its efficacy in patients with refractory neocortical epilepsy.	Snowball et al. (2019)
	AAV9	*CAMK2A*	EKC	Frontal cortex	FCD mice	Reduced seizure frequency, but no behavioural improvements.	
	AAV2/9	*CAMK2A*	EKC	Hip	KA (i.p) rats	AAV injection resulted in reduced seizure frequency and duration.	
	AAV9	*cFos*	EKC	Hip	PTZ (i.p) mice	Activity-dependent expression of Kv1.1 had no effect on behaviour but attenuated seizure severity and improved survival after repeated PTZ injections.	Qiu et al. (2022)
					Amygdala KA amygdala mouse model	Treatment resulted in reduced spontaneous seizure numbers and spike activity.	
Neuropeptide Y and receptors	AAV1/2	*NSE*	NPY	Hip	KA (Hip) rats Electrical kindling rats	Injections abolished status epilepticus and significantly delayed kindling acquisition.	Richichi et al. (2004)
	AAV1/2	*NSE*	NPY2R	Hip	KA (s.c) rats Electrical kindling rats	AAV treatment reduced seizure severity and progression, improved survival and reduced latency to status epilepticus.	Woldbye et al. (2010)
	AAV1/2	*NSE*	NPY2R/NPY	Hip	Hip KA rats Electrical kindling rats	AAV injection resulted in increased kindling- threshold after discharge while reducing seizure frequency and duration.	Ledri et al. (2016), Melin et al. (2019)
	AAV1/2	*NSE*	NPY5R/NPY	Hip	KA (s.c) rats.	Administered treatment increased latency to status epilepticus and reduced seizure severity.	Gotzsche et al. (2012)
p38γ	AAV PHP.B	*hSyn*	*MAPK12 -D179A*	*IV*	*Scn1a* ^*+/*−^ and pilocarpine (i.p) mice	Treatment resulted in Increased p38γ expression and phosphorylation of tau at T205. This restored neuronal firing patterns, improved EEG profiles and behaviour, whilst reducing seizure severity, and increasing survival.	Morey et al. (2022)
SCN1A	AAV9	*RE* ^*GABA*^	EXT101 eTF^SCN1A^	ICV	*Hemizygous Scn1a* ^*+/*−^ and R1407X mice	Increased NaV1.1 expression in GABAergic interneurons extended survival and reduced spontaneous seizures in a DS mouse model. This gene therapy is approved for clinical trial phase 1/2-ENDEAVOR.	Tanenhaus et al. (2022)
Neuro D1	AAV9	*hGFAP1.6*	NeuroD1	Hip	Pilocarpine (i.p) rats	Treatment converted astrocytes into GABAergic interneurons, protecting against neuronal loss, reducing spontaneous seizures, and rescuing behavioural performance.	Zheng et al. (2022)

AAV, adeno-associated virus; AAV PHP.B, modified neurotrophic variant of AAV9; CAMK2A, Calcium/Calmodulin-Dependent Protein Kinase II, alpha promoter; CMV, cytomegalovirus promoter; c-fos stimuli responsive immediate early gene promoter; Del., delivery route; DS, dravet syndrome; eTF, engineered transcription factor; FCD, focal cortical dysplasia; hGFAP1.6, human glial fibrillary acidic protein 1.6 kb promoter region; HIP, hippocampal; hSyn, human synapsin promoter; ICV, intracerebroventricular injection; i. p., intraperitoneal injection; IV, intravenous injection; KA, kainic acid; LV, lentivirus; NSE, Neuron-Specific Enolase promoter; PTZ, pentylenetetrazol; RE^GABA^, GABAergic inhibitory interneurons-selective regulatory element; RMC, right motor cortex; s.c., subcutaneous injection.

## Strategy: gene supplementation

Gene supplementation is a relatively direct approach in which a functional copy of a gene is introduced into a cell to compensate for the absence or malfunction of the native gene. This strategy is particularly well-suited to address epileptic conditions characterized by underlying loss-of-function genetic mutations (see Table 1). Yet it faces a notable challenge: the limited packaging capacity of viral vectors. A key example is the ***SCN1A*** gene–just over 6 kb in size, the *SCN1A* sequence is too large to be delivered via an AAV vector. To overcome this, researchers have used a high-capacity AdV vector to deliver the full *SCN1A* coding sequence under the control of the ubiquitous CAG promoter for widespread expression across all cell types (Mora-Jimenez et al., 2021). DS mice treated via intracerebral injection showed normalized EEG parameters, improved survival, greater resistance to hyperthermia-induced seizures, and some behavioural improvements (Mora-Jimenez et al., 2021). This approach was then further refined by replacing the CAG promoter with a GABAergic neuron-specific hybrid DP3V promoter, resulting in improved survival and resistance to hyperthermia-induced seizures in mutant *Scn1a* mice (Ricobaraza et al., 2023). GABAergic neurons were selectively targeted because the loss of interneuron excitability is believed to play a driving role in DS (Ogiwara et al., 2013). Thus, selective targeting of interneurons can (depending on the approach) potentially increase both therapeutic safety and efficacy. Most recently, multiple dual-AAV systems have been developed to overcome the packaging restriction of AAVs (Mich et al., 2025; Lin et al., 2026). The first approach involved the interneuron-specific expression of a modified *SCN1A* sequence, divided into two segments at Cys 1050 to fit within two independent AAV vectors, with intein-mediated scarless reconstitution of the two-halves after translation (Mich et al., 2025). The second approach on the other hand, employed neuron-specific expression of the *SCN1A* sequence split at nucleotide c.3771 into two independent AAV vectors, and leveraged Cre/lox-mediated intermolecular DNA recombination at the episomal level via loxP sites prior to transcription (Lin et al., 2026). While the delivery routes differ slightly (intracerebroventricular vs. retro-orbital, respectively), co-injection of the dual vectors into DS mice at birth provides significant protection against postnatal mortality, hyperthermia-induced seizure (Mich et al., 2025; Lin et al., 2026), and has been shown to rescue hyperexcitability in hippocampal neurons and reduce seizure-related electrocorticography (ECoG) activity patterns in DS mice (Lin et al., 2026).

**Glycosylphosphatidylinositol (GPI)-anchored proteins**, which uniquely attach to the outer surface of cell membranes using a GPI “hook” and thereby facilitate important cellular processes such as fertilization, development, and immune responses (Kinoshita, 2020). Numerous genes are involved in the biosynthesis and modification of these GPI-anchored proteins, and variations in these genes can cause GPI deficiencies (Sidpra et al., 2024). The ***PIGA*** gene encodes an essential catalytic component involved in GPI biosynthesis. Variants in *PIGA* are associated with neurological abnormalities, including profound developmental delay and epileptic seizures. The gene is X-linked, meaning that only male progeny who receive a variant allele are affected (Bay et al., 2020). Researchers have reported a gene-supplementation strategy to treat mice with CNS-specific *Piga* knockout (Murakami et al., 2024). The highly neurotrophic PHP.eB AAV serotype was used to deliver the human *PIGA* sequence under the control of the ubiquitous CAG promoter. Mice treated at birth showed significantly extended lifespans, with hemizygous females exhibiting longer survival than hemizygous males (Murakami et al., 2024). Treated females were healthy enough to participate in some behavioral tests, and no spontaneous seizures were observed, while untreated females were too severely affected to allow direct comparison. However, treatment did not improve brain structural abnormalities, suggesting that not all defects are (easily) reversible (Murakami et al., 2024).

**PIGO** is a transferase that attaches GPIs to proteins and is therefore involved in the late stages of GPI-anchor synthesis. The clinical spectrum associated with *PIGO* mutations overlaps with *PIGA* deficiency, including developmental delays and seizures (Nakamura et al., 2014; Tanigawa et al., 2017). However, overexpression of PIGO is predicted to have detrimental effects on other GPI- proteins (Hunt et al., 2023). Researchers have therefore developed a novel method known as homology-independent targeted integration assisted by low-level transgene expression (HITI-TE) to achieve transgene expression at levels comparable to those of the endogenous gene (Kuwayama et al., 2022). This involved targeted insertion of the full *Pigo* sequence into the 5′UTR of the endogenous locus (with expression controlled by the endogenous promoter) via DNA-editing enzyme CRISPR-Cas9 and a relevant gRNA (Kuwayama et al., 2022). Pigo expression thus resulted from both the integrated *Pigo* cDNA sequence and from the extra-chromosomally maintained guide RNA (gRNA) AAV, as it contained the full *Pigo* cDNA coding sequence. Both AAVs were administered via intravenous injection at birth, and treated mice showed improved growth defects, motor performance and importantly, no overt seizures (Kuwayama et al., 2022).

Together, these examples highlight the clinical potential of gene supplementation which could be applied to a wide range of genetic conditions. This strategy could potentially circumvent the need to design personalized therapies to correct each patient’s unique individual pathogenic mutation - or multiple mutations in the case of PIGO deficiency (Morren et al., 2017). However, in the case of dominant-negative mutations, where the mutant protein can interfere with the function of a protein translated from the unaffected allele, this approach may not prove successful, although such examples are considered rare in epilepsy (Orhan et al., 2014). Whilst these studies are promising, it remains unclear whether gene supplementation can effectively address all symptoms, particularly those resulting from impairments in early embryonic processes. It is possible that introducing a correct copy of a gene may, in some cases, only prove mildly effective. Ideally, the continued development of novel treatment approaches will help establish the best strategies for maximizing therapeutic efficacy for each unique condition.

## Strategy: gene editing

The discovery of clustered regularly interspaced short palindromic repeats (CRISPR) has transformed genetic engineering, offering a powerful tool to modify genes permanently (Deltcheva et al., 2011; Jinek et al., 2012). However, several challenges must be overcome to enable the broad application of this technology to neurological conditions. These includes safe and efficient delivery of CRISPR to the brain, immune activation in response to CRISPR components and off-target gene editing (Gough and Gersbach, 2020; Hunt et al., 2023). Considering that over 800 genes are associated with DEEs alone (Specchio et al., 2024), the logistics of creating thousands of bespoke gene therapies to correct relatively rare mutations is a monumental task. Ethical considerations and regulatory constraints may further complicate the pathway to clinical application. Despite these challenges, CRISPR technology holds immense promise for epilepsy treatment (Pacesa et al., 2024). Therefore, whilst the long-term effects of gene editing still need to be fully elucidated, continued advancements will certainly bring us closer to realizing the full potential of CRISPR (see Table 1).

**Gene disruption**. An example of how CRISPR could be applied to epilepsy comes from a study that utilized CRISPR-associated protein 9 (Cas9) to specifically target **5-lipoxygenase (Alox5)** in neurons (Guan Q. et al., 2024). Alox5 is involved in the production of pro-inflammatory mediators, and its activity has been linked to seizure-induced neuronal damage (Mao et al., 2022). Scientists thus hypothesized that neuron-specific depletion of Alox5 would show therapeutic efficacy against seizures. To achieve neuron-specific expression, CRISPR-Cas9 was delivered using AAV9 under the control of the human synapsin promoter (hSyn), while the gRNA was driven by the RNA polymerase III promoter U6 to ensure efficient transcription (Guan Q. et al., 2024). This approach successfully disrupted the genomic sequence for Alox5, leading to a significant reduction in protein expression within neurons (Guan Q. et al., 2024). In wild-type mice, intrahippocampal injection of the AAV significantly reduced seizure severity, susceptibility as well as EEG abnormalities when later challenged with either pilocarpine (muscarinic receptor agonist) or kainic acid (glutamate receptor agonist), demonstrating the anti-convulsant effect of this therapy (Guan Q. et al., 2024). Moreover, mice that received AAV treatment after seizure induction displayed notable improvements in EEG patterns, anxiety-like behaviour, cognitive performance, and autistic-like traits when later assessed (Guan Q. et al., 2024). Histological analysis revealed reduced neuronal loss, gliosis, and mossy fiber sprouting in treated mice, suggesting a broad impact on epileptogenic pathology (Guan Q. et al., 2024). Collectively, these findings suggest that Cas9-mediated disruption of Alox5 in neurons may offer a promising therapeutic strategy for epilepsy.

**Gene upregulation**. In its original format, Cas9 functions as an endonuclease that can cleave genomic target sites. However, a catalytically inactive variant of Cas9 (dCas9) can also be utilized to modulate gene activity (Gilbert et al., 2014). For example, dCas9 can be fused to an activator or repressor and then directed to the promoter sequence of a target gene using a gRNA. This approach enables modulation of gene transcription without introducing any DNA breaks, allowing the target gene to be expressed in its native form. Using this approach, scientists have manipulated the expression of various ion channels to modulate neuronal excitability (Table 1). For example, CRISPR activation (CRISPRa) was used to direct dCas9 to the promoter region of the ***KCNA1*** gene to increase protein expression of the associated K_v_1.1 potassium channel subunit (Colasante et al., 2020a), as reduced expression or dysfunction of K_v_1.1 has been implicated in several forms of epilepsy. Here, seizures were induced via intra-amygdala injection of kainic acid followed by administration of the AAV9-based CRISPRa therapy into the right ventral hippocampus to enhance *KCNA1* expression (Colasante et al., 2020a). Treated mice showed a significant reduction in the number of seizure events and improved cognitive performance, although EEG parameters remained unchanged (Colasante et al., 2020a). In a second example, scientists utilized CRISPRa to upregulate the expression of *KCC2*, a K-Cl cotransporter critical for maintaining neuronal inhibition by regulating intracellular chloride levels (Shi et al., 2023). Dysfunction of this transporter has been strongly linked to several epilepsy subtypes. AAV was used to deliver a split-intein dCas9 system designed to target the promoter region of *KCC2*, enabling activation of the endogenous gene specifically in neurons (via a CAMK2A promoter) (Shi et al., 2023). To test this approach, mice underwent hippocampal kindling (where repeated focal stimulation of the brain is applied to enhance seizure susceptibility), followed by AAV injection into the subiculum. Upon rekindling several weeks later, treated mice showed reduced seizure severity and improved EEG measures. Interestingly, AAV treatment also conferred responsiveness to sub-clinical doses of either diazepam or valproate (Shi et al., 2023), with the greatest seizure protection observed in animals receiving both the gene therapy and ASM (Shi et al., 2023). Together, these findings demonstrate that targeted upregulation of specific ion channels is a promising therapeutic strategy for epilepsy and may help overcome drug resistance. Further, these studies highlight the versatility of CRISPRa in modulating endogenous gene expression across various epileptic conditions.

To target more specific disease mechanisms, researchers have developed a dCas9-based system to restore **NaV1.1** sodium channel protein levels selectively in interneurons as a treatment option for DS (Colasante et al., 2020b). Here, dCas9 was fused to a transcriptional activator (VP64) to promote expression of the *SCN1A* gene, which encodes NaV1.1 (Colasante et al., 2020b). Scientists used a dual AAV9-based system to express the dCas9-VP64 gene activator as well as a gRNA targeting the Scn1a promoter sequence, using the mDlx5/6 promoter to ensure selective expression in forebrain GABAergic interneurons. The AAVs were administered at birth via intracerebroventricular injection into DS mice that were hemizygous for *Scn1a*. Following treatment, mice showed greater resistance to hyperthermia-induced seizures and shorter seizure duration in EEG recordings (Colasante et al., 2020b). These findings demonstrate that dCas9-mediated gene activation can also be tailored to selectively target disease mechanisms for specific epileptic conditions. With further refinement, it may even become possible to package this system into a single plasmid, potentially leading to better co-expression of the therapeutic elements and thus more pronounced effects.

## Strategy: regulation of mRNA expression with ASOs

While the examples highlighted thus far focused on strategies to target genomic DNA sequences, an alternative approach is to target RNA transcripts. This can be achieved through a range of methodologies, such as utilising the CRISPR-Cas13 protein family which is uniquely capable of targeting RNA rather than DNA (Abudayyeh et al., 2016; East-Seletsky et al., 2016). In the context of epilepsy, there is already clinical evidence to suggest that this approach can be effective (Table 2). Specifically, Stoke Therapeutics has developed an antisense oligonucleotide (ASO) therapy, **STK-001** (Zorevunersen), to prevent the inclusion of the “poison” exon 20N in *SCN1A* RNA transcripts. Inclusion of this exon triggers nonsense-mediated decay and prevents the production of full-length mRNA (Carvill et al., 2018). Preclinical studies in mice demonstrated that STK-001 can increase full-length *Scn1a* mRNA and NaV1.1 protein expression, leading to improved survival and reduced seizure severity in a DS model (Han et al., 2020; Wengert et al., 2022). Subsequent studies that utilized the surrogate ASO-84 likewise showed strong efficacy, with restoration of action potential firing, sodium current density, and GABAergic signalling in parvalbumin-positive interneurons in DS mice (Yuan et al., 2024). These findings provided a strong rationale for clinical trials with some studies already reaching completion (NCT04442295 – ADMIRAL, LONGWING), with a phase II open-label extension study currently underway (NCT04740476 – MONARCH, SWALLOWTAIL) and a global phase 3 trial that commenced mid-2025 (NCT06872125 - EMPEROR). Early results are encouraging, suggesting substantial and durable reductions in seizure frequency along with continuing improvements in cognition, behaviour and quality of life (Meglio, 2023). In another example, Praxis Precision Medicines is currently evaluating an *SCN2A*-targeting ASO-based treatment known as **PRAX-222** (Elsunersen) in Phase1/2 clinical trials (NCT05737784 - EMBRAVE). This approach is specifically designed for the treatment of paediatric patients with *SCN2A* gain-of-function DEEs with the aim of reducing NaV1.2 protein levels. Early results are promising, validating the clinical translatability of this approach. Together, these findings demonstrate that mRNA-targeting approaches may offer a solution to address both the underlying syndrome and its associated seizures.

The versatility of ASO technology is apparent from additional studies aimed at correcting other ion channel dysfunctions commonly associated with DEEs (Specchio et al., 2024). For example, ASO-mediated suppression of sodium channel NaV1.6 (encoded by *SCN8A*) has been shown to counteract gain-of-function mutations that drive seizure activity, leading to improved survival, normalized EEG recordings, and enhanced motor performance in *Scn8a* mutant mice (Lenk et al., 2020). Notably, this ASO also demonstrated efficacy in other epilepsy models, including hemizygous *Scn1a* DS mice, mutant *Kcnq2* mice, and mutant *Kcna1* mice (Lenk et al., 2020; Hill et al., 2022). Furthermore, an ASO targeting sodium-activated potassium channel K_Na_1.1 (encoded by *KCNT1*, which is overactive in certain epilepsies) reduced neuronal hyperexcitability and improved outcomes in *Kcnt1* mutant mice, as well as prolonging survival in mutant *Scn8a* and hemizygous *Scn1a* mice (Burbano et al., 2022; Hill et al., 2023). Collectively, these findings underscore the broad therapeutic potential of ASOs in treating epileptic disorders.

Using a novel approach to target RNA splicing, scientists have also demonstrated the therapeutic benefits of ASOs to modulate protein levels of synaptic **Ras GTPase-activating protein 1 (SynGAP1)** (Cao et al., 2023), a protein located at the excitatory synapse and crucial regulator of neuronal excitability (Jeyabalan and Clement, 2016). Loss or reduced function of SynGAP1 is associated with epileptic encephalopathy (Vlaskamp et al., 2019) as well as autism spectrum disorder (ASD) and other behavioural, social, and sensory challenges (Mignot et al., 2016). Restoration of SynGAP1 protein levels in adult hemizygous mice via a Cre-recombinase system improved seizure thresholds and rebalanced neural activity, and reversed deficits in brain function (Creson et al., 2019). However, whilst increasing SynGAP1 protein levels appears to be a valid approach for managing this condition, packaging the SynGAP1 gene sequence (∼4 kb) into an AAV vector presents significant challenges. Therefore, rather than focusing on overexpressing the SynGAP1 sequence, scientists developed an approach to target the alternative 3′ splice site (A3SS) in intron 10 of SynGAP1, a region responsible for inducing nonsense-mediated mRNA decay (Yang R. et al., 2023; Dawicki-McKenna et al., 2023). In proof-of-concept studies, the team first used CRISPR Cas9 to genetically delete the A3SS in mice. This led to an upregulation of SynGAP1 protein in edited mice and effectively alleviated deficits caused by SynGAP1 hemizygosity (Yang R. et al., 2023). The team then designed ASOs, known as a splice-switching oligonucleotides, to target the A3SS sequence of SynGAP1. These were designed to bind to pre-mRNA as steric blockers, thereby redirecting splicing (Zhang, 2024). Patient-derived iPSCs and human cerebral organoids treated with the lead ASO showed suppression of mRNA decay and increased SynGAP1 protein expression (Yang R. et al., 2023). Whilst these findings highlight the potential benefits of targeting transcript splicing, it remains to be determined whether treating SynGAP1disorders with this specific ASO has any effect on seizures, and key questions regarding dosing and brain penetrance remain unanswered.

One final ASO approach of interest involves targeting an antisense transcript for the treatment of Angelman syndrome (Dindot et al., 2023). This disorder is caused by mutations that disrupt the maternal allele of the **ubiquitin-protein ligase E3A (*UBE3A*)** gene (Matsuura et al., 1997). Clinically, this leads to neurological symptoms such as developmental delay, motor and cognitive deficits as well as epilepsy (Lossie et al., 2001; Milazzo et al., 2021). One treatment strategy being explored involves reactivating the normally repressed paternal *UBE3A* allele, whose expression is customarily inhibited by a *UBE3A* antisense transcript (Elgersma and Sonzogni, 2021). A variety of strategies have been applied to reduce expression of the *UBE3A* antisense transcript, including the use of ASOs, Cas9, zinc finger nucleases and a chemically modified-gapmer ASO (single-stranded RNA-DNA-RNA molecules designed to induce cleavage of a target RNA) (Milazzo et al., 2021; Meng et al., 2015; Bazick et al., 2026; Wolter et al., 2020). These interventions have successfully reinstated paternal *UBE3A* expression, restored protein levels and improved neurological deficits in mouse models of Angelman syndrome. In fact, this success led to a Phase 1/2, dose-escalating clinical trial (NCT04259281) followed by a Phase 3 efficacy trial (ASPIRE) run by Ultragenyx Pharmaceutical Inc., which aims to assess the safety and tolerability of the ASO **GTX-102** in individuals with Angelman syndrome (Dindot et al., 2023).

## Strategy: regulation of mRNA expression with AAVs

**MicroRNAs (miRNAs)** are small (∼21 nucleotides), endogenous, non-coding, single-stranded RNA molecules that regulate gene expression by modulating post-transcriptional processes (Bajan and Hutvagner, 2020). They primarily act as negative regulators of gene expression by guiding a silencing complex to a target transcript (O'Brien et al., 2018), although some have been reported to upregulate gene expression (Vasudevan, 2012). In humans, miRNAs are processed from a primary precursor transcript via a two-step process (Shukla et al., 2011). For this reason, miRNAs require intracellular processing before becoming therapeutically active, whereas ASOs are chemically synthesized and can act immediately upon administration, without the need for cellular maturation.

Several miRNAs have been implicated in epileptogenesis (Abdel Mageed et al., 2024; Sonawane et al., 2024) and their dysregulation has been shown to impact key processes, including synapse development (e.g., miR134) (Morris et al., 2019) and synaptic plasticity (e.g., miR125, miR129) (Muddashetty et al., 2011; Rajman et al., 2017). To manipulate endogenous miRNA activity, two common strategies are employed: antagomiRs (antisense inhibitors that suppress miRNA function) and agomiRs (synthetic mimics that enhance miRNA function) (Brennan and Henshall, 2020). In the context of clinical application, these oligonucleotides only offer temporary effects, do not provide long-term genetic modifications, and share similarities with ASOs in that crossing the BBB remains a significant challenge. Scientists have therefore utilized AAVs as delivery vectors to enhance the therapeutic potential of miRNA-based interventions.

**miR-335-5p**. Using genome-wide screening, miR-335-5p was identified as a miRNA of interest in epilepsy (Heiland et al., 2023). Scientists observed that treating mice with an antimiR to specifically inhibit miR-335-5p increased neuronal excitability and seizure vulnerability. Interestingly, miR-335-5p was the only miRNA that was found to be altered in plasma samples from treatment-resistant epilepsy patients, highlighting its potential role in refractory epilepsy (Heiland et al., 2023). To explore its therapeutic potential, an AAV was designed to express the primary transcript for miRNA-335-5p under the control of the hSyn promoter (AAV9-pri-miR-335), with the aim of upregulating miR-335-5p expression. Following hippocampal AAV injection and seizure induction via pentylenetetrazol (PTZ, GABA-A receptor antagonist), mice showed reduced seizure severity and improved survival (Heiland et al., 2023). Post-mortem analysis confirmed increased miR-335-5p expression which coincided with a downregulation of several sodium channel subtypes after seizure induction. These findings support a role for miR-335-5p in the homeostatic regulation of neuronal excitability (Heiland et al., 2023). Although this strategy contradicts the therapeutic goal for DS (as it decreases sodium channel expression), targeting miR-335-5p still offers a potential therapeutic approach for other epileptic conditions. Furthermore, it provides evidence that modulating the expression of miRNA sequences is a promising avenue for epilepsy subtypes characterised by miRNA dysregulation, such as refractory epilepsy and temporal lobe epilepsy (Ghafouri-Fard et al., 2022).

**Anti-ADK siRNA**. In the previous examples, neurons were the primary cellular target. However, there is significant evidence to suggest that astrocyte dysfunction also plays a significant role in epileptogenesis (Vezzani et al., 2022). For example, patients with mesial temporal lobe epilepsy exhibit upregulation of adenosine kinase (ADK), an enzyme predominantly expressed in astrocytes. Increased ADK levels cause a reduction in adenosine - a neuromodulator with anticonvulsant properties - thereby promoting neuronal hyperexcitability and seizure activity (Aronica et al., 2011; Gouder et al., 2004). Building on these findings, scientists developed an AAV that expresses an anti-ADK short-interfering RNA (siRNA) under the control of the astrocyte-specific GFAP promoter (Young et al., 2014). It should be noted that while siRNAs and miRNAs share many similarities; siRNAs have a single mRNA target and are therefore more specific (Lam et al., 2015). Treated animals received bilateral hippocampal AAV infusions either before or after kainic acid-induced seizures. Pre-treatment reduced seizure activity, while post-treatment decreased hippocampal cell loss—demonstrating neuroprotective effects in both paradigms (Young et al., 2014). This study therefore demonstrates the therapeutic potential of targeting astrocytes for the treatment of epilepsy.

**Anti-Dnm1a siRNA**. siRNAs have also been successfully utilized to target neuronal processes linked to epilepsy. For example, AAV has been used to deliver siRNAs targeting a specific pathogenic dynamin-1 isoform, known as ***Dnm1a***. Dominant-negative mutations in the *DNM1* gene are associated with the severe developmental epileptic encephalopathyies Lennox-Gastaut syndrome and infantile spasms (von Spiczak et al., 2017). Dynamin mutant mice treated with this therapeutic AAV just after birth showed decreased seizure-related lethality, elimination of ataxia and improved growth and development (Aimiuwu et al., 2020), highlighting the broad relevance of targeting mRNA transcripts with AAV-based therapies for epilepsy.

**Anti-*grik2* siRNA**. A subclass of ionotropic glutamate receptors known as kainate receptors plays a major role in regulating neuronal excitation and has been implicated in mediating excitotoxicity in epilepsy (Hansen et al., 2021; Vincent and Mulle, 2009). The kainate receptors form via tetrameric assembly of subunits GluK1 through to GluK5, and it is the GluK2 subunit in particular that is thought to play a key role in epileptic pathophysiology (Peret et al., 2014). In support of this, gain-of-function mutations in the *GRIK2* gene that encodes GluK2 are associated with epilepsy in humans (Stolz et al., 2021). Researchers therefore developed an AAV9-based therapy to express a synthetic anti-*grik2* siRNA under the control of the hSyn promoter, with the aim of reducing GluK2 expression (Boileau et al., 2023) (Table 2). After seizures were induced with pilocarpine, the AAV was injected directly into the hippocampus of mice. The treatment achieved a GluK2 knockdown of approximately 30%, resulting in reduced hyperlocomotion and seizure frequency in mice. Interestingly, the therapy also suppressed epileptiform activity in hippocampal slices from patients with temporal lobe epilepsy (Boileau et al., 2023). Building on these results, a clinically optimized sequence containing two *GRIK2*-targeting siRNAs was developed and again demonstrated efficacy in pilocarpine-treated mice (Baudouin et al., 2024). UniQure, Inc. is currently conducting a phase I/IIa clinical trial (NCT06063850) specifically in adult patients with unilateral refractory mesial temporal lobe epilepsy to evaluate the safety, tolerability, and efficacy of a *GRIK2*-silencing AAV known as **AMT-260**.

## Strategy: therapeutic gene expression

In many cases of epilepsy, no obvious underlying cause can be identified, limiting the suitability of certain therapeutic approaches, such as gene editing. Instead, these cases may be better addressed with genetic medicines that modulate mechanisms that are broadly associated with seizure activity. For example, targeting specific ion channels, receptors, or neurotrophic factors may be beneficial in these instances. Below, we discuss various potential therapeutic candidates in more detail (Table 3).

**Potassium channel Kv1.1**. Potassium channels are of significant interest in epilepsy due to their crucial role in regulating neuronal excitability. Moreover, mutations in potassium channel genes are associated with several genetic epilepsy syndromes. Interestingly, targeting specific potassium channels has been shown to have therapeutic benefits (Wykes et al., 2012; Snowball et al., 2019; Qiu et al., 2022). One notable example involves the Kv1.1 channel. Lentiviral delivery of the *K*_*v*_ *1.1* sequence under the control of the ubiquitous CMV promoter in rats challenged with tetanus neurotoxin (rodent model of focal neocortical epilepsy) suppressed seizure activity, preventing not only the development of epilepsy but also diminishing seizures in animals with established disease (Wykes et al., 2012). This approach was further refined via the introduction of a mutation in the K_v_1.1 sequence to accelerate channel recovery after inactivation, known as **engineered potassium channel (EKC)** (Snowball et al., 2019; Almacellas Barbanoj et al., 2024). Delivery of this construct via AAV2/9 reduced both the frequency and duration of established seizures in kainic acid treated rats and genetically modified focal cortical dysplasia mice (Snowball et al., 2019). More recently, researchers explored the use of a cFos promoter to drive EKC expression, to determine whether activity-dependent promoters could be effectively used to decrease neural excitability in a closed-loop manner (Qiu et al., 2022). Following seizure induction via intra-amygdala injection of kainic acid, treated mice demonstrated a significant decrease in the number of seizures as well as a normalization of EEG recordings (Qiu et al., 2022). In a second model, the gene therapy was also found to increase the survival of mice injected with a lethal dose of PTZ (Qiu et al., 2022). Interestingly, the researchers also assessed LV-based delivery of the gene therapy in cortical organoid cultures. Whilst untreated organoids exhibited neuronal excitability following treatment with 4-aminopyridine and picrotoxin, gene therapy-treated organoids showed suppression of hyperexcitability, supporting the therapeutic potential of this approach in a human-relevant system (Qiu et al., 2022). Based on this data, a non-integrating lentiviral **EKC** therapy is currently being assessed in a phase I/IIa clinical trial (NCT04601974) for patients with refractory neocortical epilepsy who are being evaluated for surgical intervention. Together, this research demonstrates the clinical potential of targeting potassium channels as a treatment strategy for epilepsy.

**Neuropeptide Y** (NPY) is a naturally occurring neuropeptide distributed globally throughout the brain (Gotzsche and Woldbye, 2016; Vezzani and Sperk, 2004). Alterations in NPY and its receptors are often observed in brain regions crucially implicated in the onset and progression of seizures (Wickham et al., 2019). Elevated NPY levels are thought to suppress presynaptic glutamate release, thereby regulating synaptic transmission and contributing to seizure control (Colmers and Bleakman, 1994). It was therefore proposed that elevating NPY levels could mitigate seizures and epileptogenesis (Cattaneo et al., 2021) (Table 3). To investigate this, AAV1/2 vectors were used to express NPY under the control of a neuron-specific enolase (NSE) promoter (Richichi et al., 2004). The AAV was delivered into the rat hippocampus followed by seizure induction through intracerebroventricular administration of kainic acid. Treated rats demonstrated a significant reduction in both seizure frequency and duration. These findings, together with additional studies (Richichi et al., 2004; Noe et al., 2008; Foti et al., 2007; Noe et al., 2010), provide strong evidence for the therapeutical potential of NPY-based therapies for epilepsy.

NPY is known to mediate its effects in the brain through binding to the Y1, Y2 or Y5 G-protein coupled receptors (Reichmann and Holzer, 2016). Interestingly, while Y2 and Y5 receptors are thought to play a role in mediating the seizure-suppressing effects of NPY, activation of the Y1 receptors is thought to facilitate seizure activity (Cattaneo et al., 2021). Therefore, overexpression of NPY may potentially lead to both inhibition as well as promotion of seizure activity - depending on receptor engagement. To address this, scientists have explored the benefits of selective overexpression of the **Y2 receptors** as a potential treatment strategy for epilepsy (Woldbye et al., 2010). Using AAV1/2 as well as the NSE promoter, Y2 was overexpressed in the hippocampus of a rat kindling model which significantly reduced seizure severity and progression (Woldbye et al., 2010). In the same study, Y2 overexpression was also found to significantly reduce seizure latency and improve survival following subcutaneous injection of kainic acid (Woldbye et al., 2010). Building on this, a combinatorial gene therapy approach was developed, in which NPY and Y2 were co-expressed via dual AAV delivery, resulting in significantly enhanced seizure protection in both electrical kindling and kainic acid-induced models (Ledri et al., 2016; Melin et al., 2019). Similarly, AAV-mediated overexpression of both NPY and the **Y5 receptor** showed efficacy in treating seizures, increased latency to status epilepticus and decreased seizure severity following subcutaneous administration of kainic acid (Gotzsche et al., 2012), while overexpression of the Y5 receptor alone had no significant effect (Gotzsche et al., 2012). Together, these studies demonstrate the potential of NPY–based therapies and highlight selective modulation of the NPY pathway as a promising strategy for seizure control.

**p38γ kinase**. Recent studies identified the p38γ kinase as a promising therapeutic target for epilepsy. In Alzheimer’s disease mouse models, overexpression of p38γ was shown to protect neurons from amyloid-β induced excitotoxicity (Ittner et al., 2016; Ittner et al., 2020). This neuroprotective effect was mediated via p38γ site-specific phosphorylation of the protein tau, which disrupts the formation of a postsynaptic signalling complex that drives neuronal hyperexcitation. Extending this finding to epilepsy, researchers explored the potential of AAV-mediated p38γ overexpression in epilepsy mouse models (Morey et al., 2022). Here, a constitutively active variant of p38γ was delivered using a neurotrophic PHP.B serotype under the control of the hSyn promoter. The AAV was administered at birth to hemizygous *Scn1a* DS mice, resulting in reduced seizure-induced mortality, normalized EEG profiles and improved explorative behaviour (Morey et al., 2022). In a second model, mice received gene therapy treatment either prior to or following pilocarpine-induced seizure onset (Morey et al., 2022). In both paradigms, treated mice demonstrated significantly improved survival and reduced seizure severity. Moreover, researchers found that phosphorylation of tau at threonine 205 was crucial for p38γ′s protective effects, as mice carrying a phosphorylation-defective tau mutation (T205A) did not benefit from the treatment (Morey et al., 2022). Together, these experiments demonstrate that neuronal p38γ overexpression may offer broad therapeutic benefits across various epileptic disorders.

As mentioned previously, the *SCN1A* gene exceeds the packaging capacity of AAV vectors, making it difficult to apply a gene supplementation approach to treat DS. For this reason, alternative approaches to increase Scn1a protein expression without directly delivering the full genetic sequence have been developed. One such example is **ETX101**, which utilizes AAV9 to deliver an engineered transcription factor (ETF^SCN1a^) specifically to GABAergic interneurons. This transcription factor binds to genetic regulatory regions upstream of *SCN1A*, promoting its expression from the genomic DNA (Tanenhaus et al., 2022). Preclinical studies in hemizygous Scn1a and R1407X mutant DS mice demonstrated that treatment with ETX101 improves survival and reduces spontaneous seizures. Building on this, Encoded Therapeutics is currently conducting a Phase I/II clinical trial (NCT05419492) to evaluate the safety and efficacy of EXT101 in infants with SCN1A-positive DS.

Neuroregenerative therapies that aim to replenish lost cells are of potential therapeutic value to several disorders, including epilepsy (Bengzon et al., 2002). One such approach involves the expression of the neuronal transcription factor **NeuroD1**, which has been shown to reprogram reactive glial cells into functional neurons in mouse models of brain injury and dementia (Guo et al., 2014). To achieve direct glial to neuron reprogramming *in vivo*, researchers used AAV9 to deliver the *NeuroD1* sequence under the control of the astrocytic GFAP promoter (Zheng et al., 2022). In this study, rats were first subjected to pilocarpine-induced seizures, followed by bilateral stereotaxic injection of the AAV into the hippocampus 1 week later. Treated rats showed significant protection against pilocarpine-induced neuronal loss, and NeuroD1-converted neurons showed interneuron-like features with the ability to functionally integrate into neuronal circuits (Zheng et al., 2022). Most importantly, treated rats showed a significant reduction in the number of spontaneous seizures, normalized EEG activity, and improved behavioural performance. These findings suggest that the newly converted neurons effectively restored the inhibitory balance and suppressed seizure activity, thereby providing therapeutic benefits (Zheng et al., 2022). Interestingly, the study also highlighted that both the targeted brain region as well as the local microenvironment (i.e., seizure-induced changes) had a significant impact on determining cell fate during conversion. This underscores the need for further research to fully delineate the specific conditions under which this therapy can be applied. Nonetheless, it demonstrates the potential of gene therapy-based cellular reprogramming as a therapeutic avenue for the treatment of epilepsy.

## Refining gene therapy

As highlighted in this review, genetic medicines can be utilized in an assortment of ways to address a wide range of disease mechanisms (summarized in Figures 1, 2). Despite their promise, several challenges must be addressed before these therapies can become routine treatments. Key issues include safety, reversibility, administration, off-target effects as well as questions regarding long-term effects. Particularly concerning for epilepsy is the fact that the use of viral vectors is associated with immunogenicity and any additional neuroinflammation could potentially exacerbate symptoms. Even if therapies are successful, there is a risk that if the balance between excitatory and inhibitory signalling is shifted too far in the alternate direction this could cause a paradoxical worsening of symptoms.

One major issue associated with many genetic medicines is their irreversibility, as many result in sustained expression that cannot be modulated if adverse effects arise. To overcome this, inducible control systems are being incorporated to enable precise temporal control over gene expression (Breger et al., 2016), such as doxycycline-based expression systems. One such example employed an AAV-based third-generation doxycycline-ON system to deliver a constitutively active *Kcnk2* K+ leak channel (TREK-M), previously shown to protect against seizures by silencing hyperactive neurons (Dey et al., 2014). However, researchers observed “leaky” expression in the absence of doxycycline. Intriguingly, the absence of doxycycline triggered spontaneous seizures while the presence of doxycycline abolished them (Sullivan et al., 2023). Researchers postulated that preferential expression of TREK-M in inhibitory interneurons was responsible for seizure induction in the absence of doxycycline, while the presence of doxycycline induced expression across all infected neuronal types and thus abolished seizures. These studies highlight the importance of cell type specific targeting and the need for refined systems that can tightly control gene expression. Emerging technologies, such as the recently reported pA-regulator system, offer promising alternatives to address current challenges around leakiness and immunogenicity (Luo et al., 2024). Continued development in this area will be critical to ensure both safety and therapeutic precision.

To enhance the translational potential of emerging gene therapies, researchers are increasingly turning to physiologically relevant models that more accurately reflect human biology. Human-induced pluripotent stem cell (iPSC)-derived neurons and brain organoids offer powerful platforms to study gene therapy effects in a patient-specific context. These models enable detailed assessment of efficacy, safety, off-target effects, and cellular stress responses in human-like tissues. Brain organoids in particular have been shown to recapitulate key aspects of cortical development, synaptic connectivity, and even network-level oscillatory activity, making them well-suited to model circuit-level interventions in epilepsy (Brown et al., 2024). Moreover, iPSC-derived microglia have been shown to modulate hyperexcitability in neurons carrying epilepsy-associated mutations, further enhancing the utility and benefit of these models (Que et al., 2024). In parallel, non-human primates (NHPs) remain invaluable for evaluating vector delivery methods, biodistribution, immune responses, and behavioral outcomes, owing to their close anatomical, physiological, and functional similarity to humans (Basso et al., 2024). NHP models have been successfully used to study and investigate seizure dynamics, chemogenetic interventions, and the long-term effects of gene therapies on cognition and emotion (Sanabria et al., 2024; Yang L. et al., 2023). Their capacity to capture complex neuropsychiatric side effects, which are often difficult to assess in rodent systems, makes them particularly valuable in preclinical research. Together, these complementary platforms—when integrated with advanced imaging and electrophysiological techniques—can help to further refine therapeutic strategies, improve predictive power, and bridge the gap between rodent studies and clinical application.

Beyond technical challenges, ethical considerations must also be addressed. Manipulating brain activity at the circuit level carries the risk of unintended consequences on cognition, memory, emotion, and mood, raising concerns about potential neuropsychiatric effects (Kakroodi et al., 2025). These risks are especially pertinent when therapies involve irreversible genetic changes, broad or off-target modulation, or germline modifications. Germline editing in particular, remains highly controversial due to its heritable nature and the inability of future generations to consent. To mitigate such outcomes, rigorous preclinical testing, long-term monitoring, and the development of strategies for cell type or region-specific targeting and reversible or regulatable gene expression systems are essential (Kozlova et al., 2025). Transparent ethical review and robust informed consent processes will be critical, especially for interventions with lasting or potentially heritable effects.

Given the variety of ways in which gene therapies can be applied, it is possible that a range of tailored treatment options could be developed for individuals with epilepsy. These options may vary depending on the specific symptoms, underlying cause(s), and personal preferences. However, the current cost of gene therapies - many exceeding one million dollars per treatment (The gene, 2023) - creates a significant barrier to widespread accessibility (Rizig, 2025) and raises concerns regarding health equity and affordability. To ensure these therapies reach their full clinical potential, challenges related to manufacturing, scalability and cost-effectiveness will also need to be overcome through innovative production technologies, regulatory frameworks, and funding models (Kohn et al., 2023).

## Final comments

Strategies for genetic medicines to treat of epilepsy are diverse, ranging from direct targeting of disease-associated mutations or mechanisms to modulating protein expression or inducing overexpression of a therapeutic transgene. Admirably, as scientific hurdles are identified, scientists have consistently adapted and developed creative solutions to maximise the clinical efficacy of these therapies. While there is still progress to be made and undoubtedly more challenges ahead, the growing number of clinical trials that are already underway point to a future in which gene therapies will reshape the landscape of epilepsy treatment and bring us closer to achieving freedom from seizures. The extent to which these therapies can be utilized will largely depend on how effectively current hurdles can be addressed. At the same time, further research is needed before scientists can fully understand their long-term efficacy and determine how they influence other comorbidities commonly associated with epileptic conditions. It also remains to be evaluated whether combining gene therapies with other conventional treatment approaches could yield synergistic benefits. Ideally, with continued refinement and innovation, gene therapies will become available for a broad spectrum of epileptic conditions, giving patients access to a range of effective, long-lasting therapeutic options, regardless of disease aetiology.
